# Integrative analyses of mRNA and microRNA expression profiles reveal the innate immune mechanism for the resistance to *Vibrio parahaemolyticus* infection in *Epinephelus coioides*

**DOI:** 10.3389/fimmu.2022.982973

**Published:** 2022-08-19

**Authors:** Xifeng Qiao, Yuyou Lu, Jiachang Xu, Niuniu Deng, Wenjie Lai, Ziyi Wu, Haoran Lin, Yong Zhang, Danqi Lu

**Affiliations:** ^1^ State Key Laboratory of Biocontrol and School of Life Sciences, Southern Marine Science and Engineering Guangdong Laboratory (Zhuhai), Guangdong Provincial Key Laboratory for Aquatic Economic Animals and Guangdong Provincial Engineering Technology Research Center for Healthy Breeding of Important Economic Fish, Sun Yat-Sen University, Guangzhou, China; ^2^ Guangzhou Laboratory, Guangzhou, China; ^3^ Laboratory for Marine Fisheries Science and Food Production Processes, Qingdao National Laboratory for Marine Science and Technology, Qingdao, China; ^4^ College of Ocean, Haikou, China

**Keywords:** *Vibrio parahaemolyticus*, *Epinephelus coioides*, histopathological lesions, immune response, mRNA-seq, miRNA-seq

## Abstract

*Vibrio parahaemolyticus*, as one of the main pathogens of marine vibriosis, has brought huge losses to aquaculture. However, the interaction mechanism between *V. parahaemolyticus* and *Epinephelus coioides* remains unclear. Moreover, there is a lack of comprehensive multi-omics analysis of the immune response of grouper spleen to *V. parahaemolyticus*. Herein, *E. coioides* was artificially injected with *V. parahaemolyticus*, and it was found that the mortality was 16.7% in the early stage of infection, and accompanied by obvious histopathological lesions in the spleen. Furthermore, 1586 differentially expressed genes were screened by mRNA-seq. KEGG analysis showed that genes were significantly enriched in immune-related pathways, Acute-phase immune response, Apoptosis, Complement system and Cytokine-cytokine receptor interaction. As for miRNA-seq analysis, a total of 55 significantly different miRNAs were identified. Further functional annotation analysis indicated that the target genes of differentially expressed miRNAs were enriched in three important pathways (Phosphatidylinositol signaling system, Lysosome and Focal adhesions). Through mRNA-miRNA integrated analysis, 1427 significant miRNA–mRNA pairs were obtained and “*p53* signaling pathway”, “Intestinal immune network for IgA production” were considered as two crucial pathways. Finally, *miR-144-y*, *miR-497-x*, *novel-m0459-5p*, *miR-7133-y*, *miR-378-y*, *novel-m0440-5p* and *novel-m0084-3p* may be as key miRNAs to regulate immune signaling pathways *via* the miRNA-mRNA interaction network. The above results suggest that the mRNA-miRNA integrated analysis not only sheds new light on the molecular mechanisms underlying the interaction between host and *V. parahaemolyticus* but also provides valuable and new insights into resistance to vibrio infection.

## Introduction

According to the statistics of China Fisheries Yearbook, the farming output of grouper in 2020 has reached 192000 tons, ranking third in China’s mariculture fish, creating huge economic and social benefits. With the rapid development of intensive large-scale aquaculture in recent years, bacterial infections continue to break out and spread (1). *Vibrio parahaemolyticus*, a Gram-negative, halophilic bacterium, inhabits marine and estuarine environment (2). As the main pathogenic microorganism of marine fish, shrimp and shellfish, it not only brings huge economic losses to aquaculture, but also poses a great threat to human health (3). Previously, *V. parahaemolyticus* has been confirmed to cause acute effects in fish, such as severe bleeding, disintegration of the organs, and even death (4, 5). It is also reported that *V. parahaemolyticus* disrupts the actin cytoskeleton, promotes the release of pro-inflammatory factors and hijacks the nutrient of host cells to ensure its survival in the environment (6, 7). However, the interaction mechanism between *V. parahaemolyticus* and host has not yet been elucidated.

The innate immune system provides the first line of defense against microbial invasion (1). Thus, many fish could respond to pathogens and then eliminated pathogens (8–10). We authenticated that *Epinephelus coioides* is a susceptible host to *V. parahaemolyticus* and the spleen is of great significance in antibacterial innate immunity (11, 12). The spleen, as a crucial lymphatic tissue in the body, which plays a key role in innate and adaptive immunity (13). Therefore, the multi-omics analysis of spleen will be helpful to deeply understand the immune mechanism of *E. coioides* in response to *V. parahaemolyticus.* The mRNA sequencing (mRNA-seq) could reveal changes of differentially expressed genes, help discovering different metabolic processes and provide precise signaling pathways (14). The application of RNA-seq in fish has increased evidently, which can further determine the function of related immune genes. Transcriptome analysis of Chinese amphioxus (*Branchiostoma belcheri*) infected with *V. parahaemolyticus* indicated that there were many pathways involved in the immune response, such as bacterial infection, immune signal, apoptosis (15). It has been speculated that Complement pathway of innate immunity and hepcidin antimicrobial peptide may play important roles in the defense of *E. coioides* larvae against *Vibrio alginolyticusthe* by RNA-seq (16). MicroRNAs (miRNAs) are noncoding RNAs of 22-24 nt which could inhibit mRNA translation or degrade mRNA to regulate many physiological processes, such as metabolism, apoptosis, nervous system development, immuno-protection and cancer pathogenesis (17, 18). For example, it is reported that teleost miRNAs can promote the antibacterial resistance by regulating several immune-related pathways, including FoxO signaling pathway (19), Complement and coagulation cascades (20), and Intestinal immune network for IgA production (21). Nevertheless, there are a few studies on the mechanism of interactions between non-coding RNAs and coding RNAs in bony fishes (21–25). Compared with the analysis of single miRNAs or mRNAs, integrated analysis of mRNA-seq and miRNA-seq would more accurately determine the key genes regulating immunity and the miRNAs targeting these genes, which has become a trend and method to study the mechanism of anti-pathogen immune response.

In this study, *V. parahaemolyticus* and *E. coioides* were served as research objects to explore the interaction mechanism between pathogen and host. The mRNA-miRNA integrated analysis of the spleen of *E. coioides* infected with *V. parahaemolyticus* was performed, and several crucial pathways were screened, which may contribute to treatment of *V. parahaemolyticus* infection. The candidate miRNAs and target genes would be helpful to deserve more attention in more in-depth research underlying the antibacterial immune response in future.

## Results

### The clinical symptoms and cumulative survival of *E. coioides* after infection of *V. parahaemolyticus*

**Figure 1:**
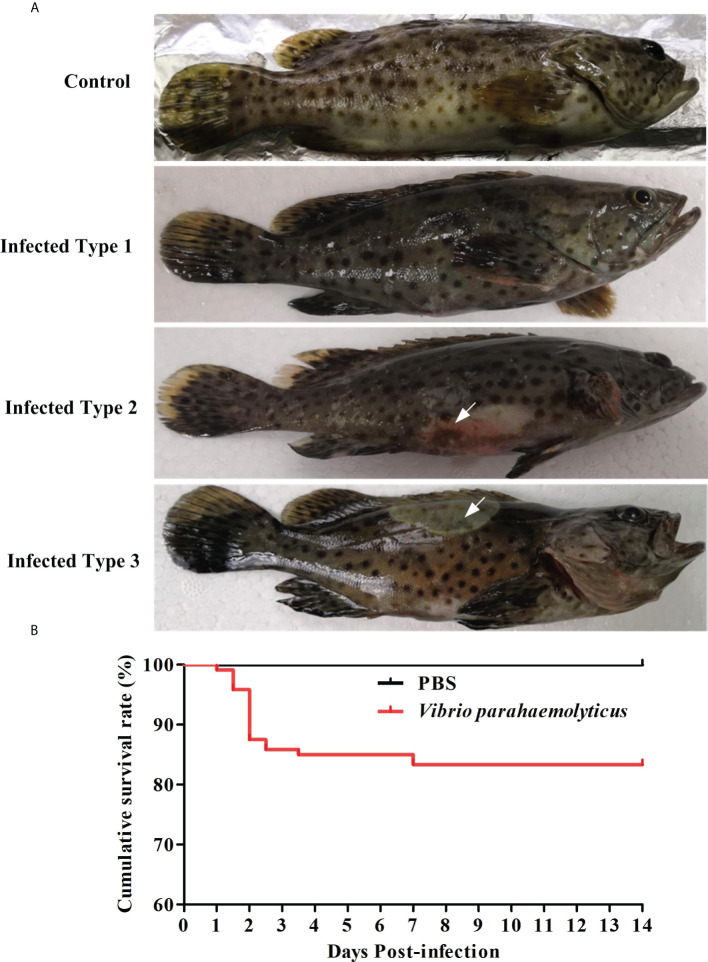
Clinical symptoms and cumulative survival of *E. coioides* challenged with *V. parahaemolyticus*. **(A)** The clinical symptoms of *E. coioides* challenged with *V. parahaemolyticus*. Infected Type 1: fish body color turned black; Infected Type 2: the swelling bleeding symptom was found near abdominal cloaca (white arrow notation); Infected Type 3: there was white patches near the dorsal fin (white arrow notation). **(B)** The cumulative survival of *E. coioides* infected with *V. parahaemolyticus* throughout two weeks. n = 60 biologically independent animals per group.

Generally, healthy *E. coioides* tended to gather at the bottom of the water tanks, with smooth body surface and vigorous swimming. During feeding, most fish swam to the upper water to compete for food. However, the fish showed isolation, slow movement and reaction, and reduced food intake on Day 1 after infection with *V. parahaemolyticus.* During infection, three distinct phenotypes were observed (**Figure 1A**), respectively the body color of many dying or dead fish turning black, the swelling bleeding symptoms near abdominal cloaca, white patches near the dorsal fin. As shown in **Figure 1B**, the survival rate of grouper was about up to 80% for 2 weeks. The death of groupers was mainly occurred on the first 3 days of infection. However, from 3 days to 7 days, the number of groupers deaths was significantly decreased. Finally, we found that there were no dead individuals from 8 days to 14 days. This result suggested that *V. parahaemolyticus* infected with grouper may be an acute infection.

### The histopathological lesions of spleen after *V. parahaemolyticus* challenge

**Figure 2:**
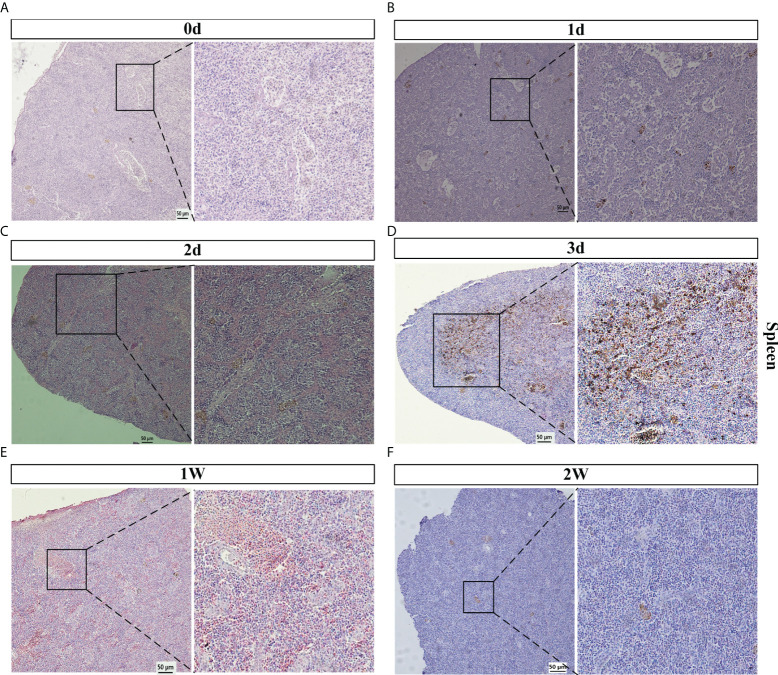
Spleen histopathology analyses of *V. Parahaemolyticus* artificially infected with *E. coioides*. **(A–F)** The spleen slice of *E. coioides* artificially infected with *V. parahaemolyticus* for 0 d (Control), 1 d, 2 d, 3 d, 1 w, 2 w. Representative images from at least three biological replicates of each time point of two groups.

In order to explore the histopathological changes in infected spleen, the morphological structures in different infection time point (1 d, 2 d, 3 d, 1 w, 2 w) were observed. As shown in **Figure 2A**, in control group, the splenic parenchyma was rich in blood sinuses, with a capsule on the surface, which is composed of white pulp, red pulp and marginal zone. The splenic cord could be obviously observed. Compared with control group, spleen pathologic changes were noted after infection, showing dilation of blood sinus, deformed white pulp and red pulp, formation of brownish nodes (These may be senescent erythrocytes, or the destruction of spleen structure and bleeding) on 1 d (**Figure 2B**). There are more brownish nodes distributed in the spleen, and it’s tough to distinguish between red pulp and white pulp on 2 d and 3 d (**Figures 2C, D**). However, compared with the early stage of infection, the lesion of spleen was significantly reduced on 1 w and 2 w (**Figures 2E, F**). This result suggested that the obvious lesions in spleen were observed in the early stages of *V. parahaemolyticus* infection.

### Quality control and expression pattern analysis of mRNA

**Figure 3:**
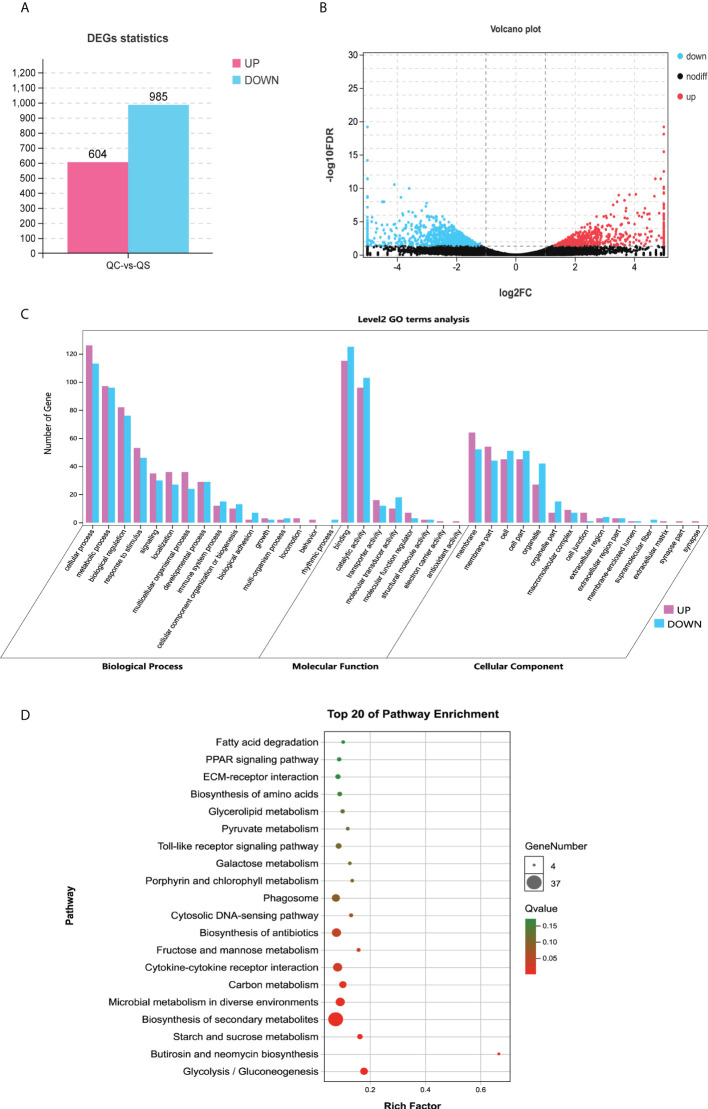
Identification and functional annotation of differentially expressed genes. **(A)** Histogram shows differentially expressed genes. Blue and red indicate decreased and increased expression, respectively. **(B)** Volcano map shows differentially expressed genes. The abscissa represents the fold change values of samples in control group/samples infected by *V. parahaemolyticus*, the vertical coordinate represents statistical test value [-lg (FDR)], the lower represent the more significant differences. Red dots represent up-regulated genes and blue dots represent down-regulated genes (FDR <0.05, |log2FC|>1), and black dots represent no significant difference genes. **(C)** GO enrichment analysis of DEGs. The abscissa indicates 3 GO categories with 39 GO terms, and the vertical coordinate indicates the number of unigenes. **(D)** KEGG pathway analysis of DEGs. The abscissa represents the ratio of DEGs to all genes annotated to the pathway and the vertical coordinate represents the pathways. The redder bubble indicates more obvious enrichment, with smaller *Q-value*. The larger bubble contains more differentially expressed genes. DEGs, differentially expressed genes; GO, Gene Ontology; KEGG, Kyoto Encyclopedia of Genes and Genomes.

To study the acute effects of *V. parahaemolyticus* challenge on *E. coioides* at the mRNA level, RNA sequencing was performed. By analyzing the quality of bases sequenced, it could be found that the ratio of Q20 or Q30 in all samples are greater than 90%, and the GC content ratio is within the reasonable range (**Table 1**). Meanwhile, the gene expression abundance of several samples was quantified by *FPKM* value, and showed basically similar. These results indicated that the gene expression obtained by sequencing was reliable. A total of 26934 unigenes were identified and subsequent differential expression analysis was determined by using edgeR, with parameters as the difference multiple | log2FC | > 1 and the false discovery rate (FDR) < 0.05. As shown in **Figures 3A, B**, a total of 1589 differential expressed genes were identified in 6 libraries, of which 604 were significantly up-regulated and 985 were significantly down-regulated.

**Table 1:** The reads information after filtering in RNA-seq.

Samples	Clean Data	HQ clean reads	Q20	Q30	GC	Total mapped	Uni-mapped
	(×10^9^ bp)	(×10^7^)	(%)	(%)	(%)	(%)	(%)
QC_1	7.842	5.155	98.84	95.95	48.11	79.30	78.66
QC_2	6.640	4.350	98.84	95.96	48.37	79.97	79.29
QC_3	5.761	3.771	98.74	95.66	48.49	76.31	75.53
QS_1	5.666	3.720	98.88	96.07	48.53	81.89	81.33
QS_2	5.254	3.442	98.74	95.64	48.37	82.42	81.83
QS_3	5.781	3.790	98.81	95.88	48.31	80.79	80.19

In order to deeply explain the biological function of differentially expressed genes, an enrichment analysis of GO terms containing three ontologies (molecular function, cellular component and biological process) was carried out. As shown in **Figure 3C**, differential expressed genes in infected group were enriched to 26 subcategories with 39 GO terms: biological progress (16 subcategories), cellular component (8 subcategories), and molecular function (2 subcategories). In the biological process category, the DEGs were mainly enriched in “cellular process”, “metabolic process”, “biological regulation” and “response to stimulus”. In the molecular function category, the DEGs were mainly enriched in “binding” and “catalytic activity”. In the cellular component category, the DEGs were mainly enriched in “cell”, “cell part”, “membrane”, “membrane part”. Since the biological progress category contains more differential expressed genes, of which the top 20 GO terms were listed in **Figure S1**. It is worth noting that the highly significant GO terms “immune system process”, “pyruvate metabolic process”, “monocarboxylic acid metabolic process”, “hematopoietic or lymphoid organ development”, “hemopoiesis” and “immune system development” (FDR < 0.05), which will provide a basis for further study on the pathogenic mechanism of *V. parahaemolyticus* to *E. coioides.*

Furthermore, a KEGG pathway enrichment analysis of differential expressed genes were performed with a *Q*-value < 0.05 and the top 20 KEGG pathways were shown in **Figure 3D**. Cytokine-cytokine receptor interaction, as an immune-related pathway, was significantly enriched. Besides, most of the DEGs were enriched in pathways associated with metabolism, i.e., Biosynthesis of secondary metabolites, Microbial metabolism in diverse environments, Butirosin and neomycin biosynthesis, Biosynthesis of antibiotics, Carbon metabolism. Interestingly, three pathways related to the Warburg effect in vertebrates were also significantly enriched, such as Starch and sucrose metabolism, Fructose and mannose metabolism, Glycolysis or gluconeogenesis.

### Quality control and expression pattern analysis of miRNA

To study the acute effects of *V. parahaemolyticus* challenge on *E. coioides* at the miRNA level, miRNA-seq was performed. After removing dirty reads, the number of clean tags of each sample in the treatment group and the control group is shown in **Table 2**. The length distribution of clean tags was shown in **Figure S2**, it could be found that the length was mainly distributed in the range of 20-23 nt and small RNAs of 22 nt in length were the most common. The tag sequences after removing other classes of small RNAs (rRNA, tRNA, snRNA, snoRNA, etc.) were aligned to the reference genome and the ratio of matched tags from six samples ranged from 74-79%. Finally, 618 known miRNAs and 550 novel miRNAs in total were identified using MIREAP_v0.2 software.

**Table 2:** The tags information after filtering in miRNA-seq.

Samples	Clean read	HQ clean reads	Clean tags	Match	Ratio	Known miRNA	Novel miRNA
	(×10^7^)	(×10^7^)	(×10^7^)	(×10^6^)	(%)		
QC_1	1.312	1.266	1.199	8.961	74.74	341	395
QC_2	1.183	1.034	98.84	7.913	76.50	340	369
QC_3	1.419	1.150	98.74	8.555	74.39	417	410
QS_1	1.253	1.097	98.88	8.329	75.91	362	389
QS_2	1.307	1.035	98.74	8.125	78.52	373	341
QS_3	1.380	1.225	98.81	9.096	74.28	383	381

**Figure 4:**
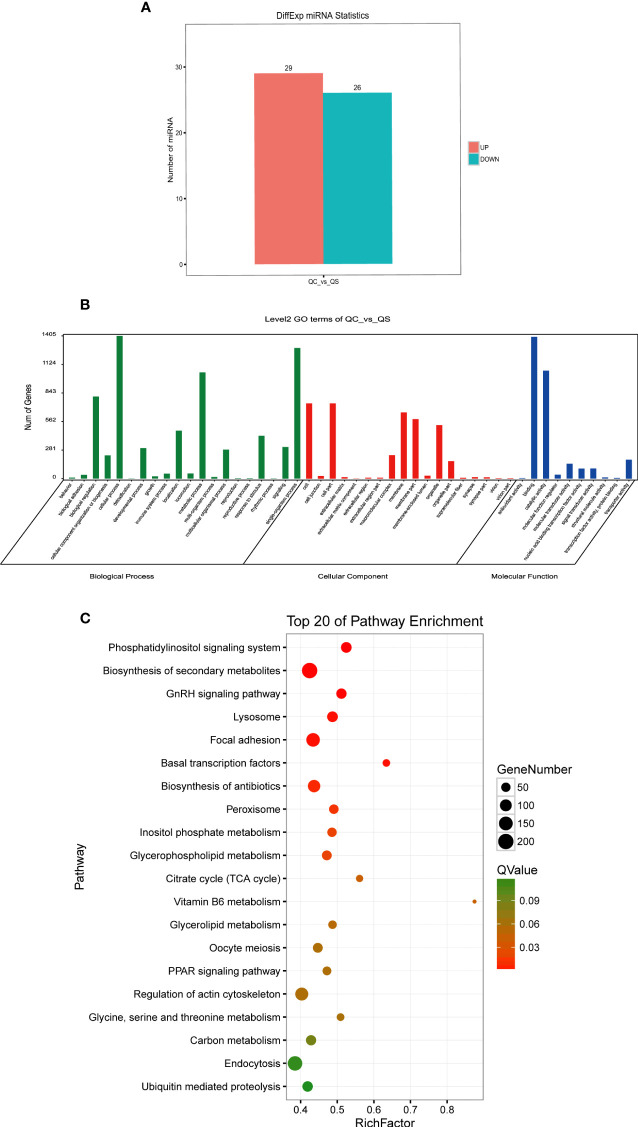
Identification and functional annotation of differentially expressed miRNAs. **(A)** Histogram shows differentially expressed miRNAs. Blue and pink indicate decreased and increased expression, respectively. **(B)** GO enrichment analysis of predicted target genes of DEMs. The abscissa indicates 3 ontologies (molecular function, cellular component and biological process) with 48 GO terms, the vertical coordinate indicates the genes count. **(C)** KEGG pathway analysis of target genes of DEMs. The redder bubble indicates more obvious enrichment, with smaller *Q-value*. Size of the point refers to the number of genes within each pathway. DEMs, differentially expressed miRNAs; DEGs, differentially expressed genes; GO, Gene Ontology; KEGG, Kyoto Encyclopedia of Genes and Genomes.

Differential expression analysis of the obtained miRNAs was performed using edgeR software and the screening criteria of DEMs were fold change ≥ 2 and *P-value* < 0.05. In this case, a total of 55 significantly different miRNAs were screened, including 29 significantly up-regulated DEMs and 26 significantly down-regulated DEMs (**Figure 4A**). The predicted target genes of DEMs were processed for further functional characterization.

As shown in **Figure 4B**, the GO enrichment analysis indicated that the predicted target genes of DEMs were clustered into three GO ontologies (molecular function, cellular component and biological process) with 48 GO terms. In the molecular function category, the target genes were mainly enriched in “binding” and “catalytic activity”. In the cellular component category, the target genes were mainly enriched in “cell”, “cell part”, “membrane”, “membrane part”. In the biological process category, the target genes were mainly enriched in “metabolic process”, “cellular process”, “biological regulation” and “single-organism process”.

Furthermore, the top 20 KEGG pathways of the predicted target genes were identified with a *Q-value* < 0.05 according to KEGG database (**Figure 4C**). Phosphatidylinositol signaling system was significant, and the pathway Inositol phosphate metabolism was also enriched. Biosynthesis of secondary metabolites, a pathway containing the largest number of target genes, was also significantly enriched in the KEGG pathway analyses of DEGs. Furthermore, Focal adhesion, a pathway associating with cytoskeleton stabilization was enriched. Meanwhile, Lysosome, a significantly enriched pathway, was concerned. Other enriched pathways were: Biosynthesis of antibiotics, Glycerophospholipid biosynthesis, Citrate cycle (TCA cycle), Basal transcription factors, GnRH signaling pathway and Peroxisome.

### Integrated analysis of mRNA-seq and miRNA-seq data

The effect of miRNA on target gene is mainly to regulate its post transcriptional level by inhibiting or silencing the expression of target gene. It has been reported that miRNA can promote protein translation through complex mechanisms, however, it is uncertain whether these are individual cases (26–28). Hence, the screening criteria of miRNA-mRNA pairs was Pearson’s correlation coefficient < -0.7 and *p* < 0.05 and a total of 1427 pairs were obtained. To further determine the function of miRNAs in the immune response of *E. coioides* against *V. parahaemolyticus* infection, the enrichment analysis of GO terms and KEGG pathways were performed on the target DEGs of DEMs.

**Figure 5:**
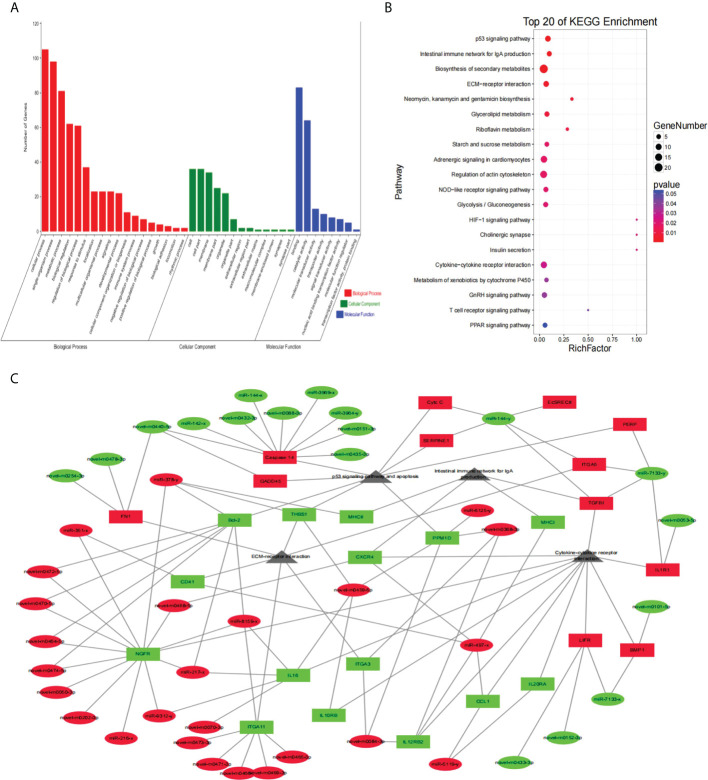
Functional annotation analysis of target DEGs of DEMs and network for miRNA-mRNA-Pathway interaction. **(A)** GO enrichment analysis of target DEGs of DEMs. The abscissa indicates 3 ontologies (molecular function, cellular component and biological process) with 39 GO terms, the vertical coordinate indicates the genes count. **(B)** KEGG pathway analysis of target DEGs of DEMs. The abscissa represents the ratio of the number of genes in the DEGs and the vertical coordinate represents the pathways. The redder bubble indicates more obvious enrichment, with smaller *P-value*. The larger bubble contains more target genes. **(C)** Network diagram for miRNA-mRNA-Pathway interaction. Ellipse represents miRNA, Round Rectangle represents mRNA, Triangle represents signaling pathway; Red color indicates up-regulated, blue color indicates down-regulated, gray color indicates no difference. DEGs, differentially expressed genes; DEMs, differentially expressed miRNAs; GO, Gene Ontology; KEGG, Kyoto Encyclopedia of Genes and Genomes.

As for the GO enrichment analysis, the target DEGs were enriched in three GO ontologies with 23 GO terms (**Figure 5A**). In the molecular function category, most of target DEGs were enriched in “binding activity” and “catalytic activity”. In the cellular component category, the target DEGs were mainly enriched in “cell”, “cell part”, “membrane”, “membrane part”. In the biological process category, the target genes were mainly enriched in “cellular process”, “metabolic process”, “biological regulation” and “single-organism process”. Moreover, the KEGG pathway enrichment analysis of target DEGs of DEMs was shown in **Figure 5B**. In the top 20 KEGG pathways, several immune-related pathways were mainly enriched, i.e., *p53* signaling pathway, Intestinal immune network for IgA production, Cytokine-cytokine receptor interaction. In addition, there are several pathways associated with metabolism, including the Biosynthesis of secondary metabolites, Neomycin, kanamycin and gentamicin biosynthesis, Glycerolipid metabolism, Riboflavin metabolism. The Starch and sucrose metabolism, and Glycolysis or gluconeogenesis were also significantly enriched in the KEGG pathway enrichment analysis. Meanwhile, two pathways related to bacterial invasion should not be ignored, i.e., ECM-receptor interaction and Regulation of actin cytoskeleton.

In order to further explore the role of miRNA-target pairs in the resistance of *V. parahaemolyticus* infection, a miRNA-mRNA-Pathway network was constructed by 80 significant pairs and four selected pathways (*p53* signaling pathway, Intestinal immune network for IgA production, Cytokine-cytokine receptor interaction and ECM-receptor interaction), showing that one miRNA could regulate multiple mRNAs, and multiple target genes were associated with several signal pathways (**Figure 5C**). Further, *miR-144-y*, *miR-497-x*, *novel-m0459-5p*, *miR-7133-y*, *miR-378-y*, *novel-m0440-5p* and *novel-m0084-3p* may be key miRNAs that play an important role in regulating immune signaling pathways. It is worth noting that scavenger receptor class F member 2 (*srec2*), as one of the key target genes, could also participate in the interaction pathway.

### The validation of sequencing results by qRT-PCR

**Figure 6:**
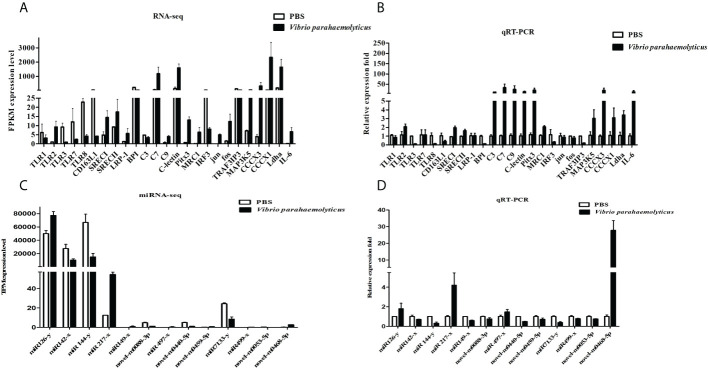
Verification of the sequencing results using qRT-PCR analysis. **(A)** Relevant pattern recognition receptors, signal molecules and cytokines in mRNA-seq. The abscissa indicates the names of the DEGs, and the vertical coordinate represents the *FPKM value*. **(B)** Relevant pattern recognition receptors, signal molecules and cytokines were verified by qRT-PCR verification. The abscissa indicates the names of the DEGs, and the vertical coordinate represents the fold change. **(C)** Selected differential expressed miRNAs were detected in miRNA-seq. The abscissa indicates the names of the DEMs, and the vertical coordinate represents the *TPM value*. **(D)** Selected differential expressed miRNA were verified by qRT-PCR. The abscissa indicates the names of the DEMs, and the vertical coordinate represents the fold change.

To verify the accuracy of mRNA-seq results, 25 differentially expressed genes were selected, such as pattern recognition receptors, signal molecules and cytokines, etc. The expression variations of DEGs were calculated by *FPKM* value (**Figure 6A**). Then the selected DEGs were verified by qRT-PCR, which showed a high degree of concordance between qRT-PCR verification and RNA-seq analysis, but there were inconsistencies in individual genes, such as complement component C3 (*c3*), toll-like receptor 7 (*tlr7*) (**Figure 6B**).

Similarly, 13 differentially expressed miRNAs were selected to verify the accuracy of miRNA-seq results by qRT-PCR. The results showed that the verification of DEMs by qRT-PCR was highly consistent with the results of miRNA-seq, except for a few miRNAs including *miR-419-x*, *novel-m0459-5p* and *miR-499-x* (**Figures 6C, D**).

## Discussion

In this study, the immune mechanism of *E. coioides* infected with *V. parahaemolyticus* was explored. Consistent with previous studies of *V. parahaemolyticus* infection in bony fish (5, 29), we found that death principally occurred in the early stage of infection with obvious tissue lesions. Furthermore, DEGs and DEMs from spleen transcriptomics of *E. coioides* were identified, and a miRNA-target-Pathway network was constructed. Basing on these results, several crucial pathways and miRNA-mRNA pairs were explored, which sheds new insights into resistance to vibrio infection.

Through the mRNA-seq analysis, 604 upregulated and 985 downregulated DEGs were screened in six libraries. In the GO enrichment analysis, the DEGs were annotated to 39 GO terms and the GO terms “metabolic process”, “immune system process” and “response to stimulus” indicated that the infection may affect host metabolism, and the host may resist the stimuli by initiating immune response. As for KEGG pathway analysis, the four pathways of “Cytokine-cytokine receptor interaction”, “Starch and sucrose metabolism”, “Fructose and mannose metabolism” and “Glycolysis or gluconeogenesis” were focused on. It is believed that Cytokine-cytokine receptor interaction pathway contributes to neutrophil mediated phagocytosis and extracellular trap formation, and the immune response is dominated by infiltrating neutrophils (30, 31). Cytokines are produced by immune-related cells, which can regulate the immune response of the body, including interleukins, lymphokines, monokines, interferons, and chemokines (32). After infected with *Yersinia pseudotuberculosis*, the highest infection-induced immunomodulatory genes were those of the major proinflammatory cytokines: interleukin-1 alpha (*il1a*), interleukin-1 beta (*il1b*), interleukin-6 (*il6*), interleukin-17F (*il17f*) and interferon gamma (*ifng*) (31). A study in *E. coioides* showed that the Cytokine-cytokine receptor interaction pathway was participated in the immune process and *il6*, *il1b* and *il1r2* were found to play key roles during the defense against* Pseudomonas plecoglossicida* (33). In the Cytokine-cytokine receptor interaction pathway, several genes of chemokines, interleukins and cytokine receptors were enriched. They are C-C motif chemokine ligand 1 (*ccl1*), C-C motif chemokine ligand 2 (*ccl2*), C-C motif chemokine ligand 3 (*ccl3*), C-C motif chemokine ligand 4 (*ccl4*), C-C motif chemokine ligand 28 (*ccl28*), C-C motif chemokine ligand 14 (*ccl14*), *il6*, *il1b*, interleukin-11 beta (*il11b*), interleukin-2 (*il2*), interleukin-16 (*il16*), interleukin-6 receptor (*il6r*), interleukin-7 receptor (*il7r*), interleukin-18 receptor accessory protein (*il18rap*), interleukin 10 receptor subunit beta (*il10rb*), C-X-C motif chemokine receptor 3 (*cxcr3*), C-C motif chemokine receptor 3 (*ccr3*) and C-X-C motif chemokine receptor 4 (*cxcr4*). Therefore, we propose that numbers of cytokines and cytokine receptors were involved in the immune process to resist *V. parahaemolyticus* infection.

Several pathways related to the Warburg effect cannot be ignored, including Starch and sucrose metabolism, Fructose and mannose metabolism, Glycolysis or gluconeogenesis. The Warburg effect was first proposed in tumor cells (34) and it was later determined that the effect plays an key role in replication of many virus, such as human papillomavirus (HPV) (35) and severe acute respiratory syndrome coronavirus 2 (SARS-CoV-2) (36). Briefly, the Warburg effect can help cancer cells or viruses escape apoptosis and host immune response by enhancing anaerobic respiration under aerobic conditions, and also perform efficient glycolysis to produce abundant energy and nutrition to promote cancer cell proliferation or virus replication (37, 38). And that, *V. parahaemolyticus* could use T3SS1 to utilize the nutrient of host cells and avoid phagocytosis by immune cells responding to proinflammatory signals at the site of infection (6). Although there are few studies on this effect in bacteria, combined with the pyruvate metabolic process GO term and the related pathways enriched by KEGG enrichment analysis, we boldly speculate that *V. parahaemolyticus* may actualize its proliferation and escape immune response by affecting the metabolism of the host.

Because our genome annotation work is still in progress, many poorly annotated DEGs do not appear in the enrichment pathways of annotated genes. Interestingly, through sequence alignment and subsequent sorting of these genes, it was found that they were enriched in three immune-related pathways, i.e., Acute-phase immune response, Apoptosis and Complement system. Therefore, we speculate that the immune response against *V. parahaemolyticus* was concentrated in the early stage of infection and belonged to acute infection. In Acute-phase immune response pathway, genes, serum amyloid A3 (*saa3*), hepcidin 3 (*hepc3*), pentraxin 3 *(ptx3)*, which encode SAA3, HEPC, PTX3, respectively, were up-regulated. SAA3, a major acute-phase protein, which released in response to inflammation (39) and produced more ubiquitously by intestine and lung (40). LPS, a component of Gram-negative bacteria, significantly enhanced the expression of SAA3 in mouse colonic epithelial cells, rather than the expression of SAA1 or SAA2 (41). HEPC, as an acute-phase protein, is expressed immediately after tissue injury or bacterial infection. In teleost, hepcidin regulates ion balance and innate immune response. When stimulated by poly I:C, iron dextran, bacteria, or LPS, the expression of hepcidin is up-regulated (42). *Hepcidin3*, a cysteine-rich hepcidin isoform gene, was identified in *E. coioides*, which could respond to the immune response caused by *Staphylococcus aureus* and *Pseudomonas stutzeri* (43). PTX3 is a pattern-recognition protein that is rapidly produced by a variety of cells under the stimulation of inflammatory factors and exogenous microorganism (44). Many studies have found that the expression of PTX3 increased after infection with *Pseudomonas aeruginosa* (45) or *Neisseria meningitidis* (46). Based on the above results, we deduced that SAA3, HEPC and PTX3, these acute-phase proteins, participated in the Acute-phase immune response pathway and contributed to the defense against *V. parahaemolyticus* infection.

**Figure 7:**
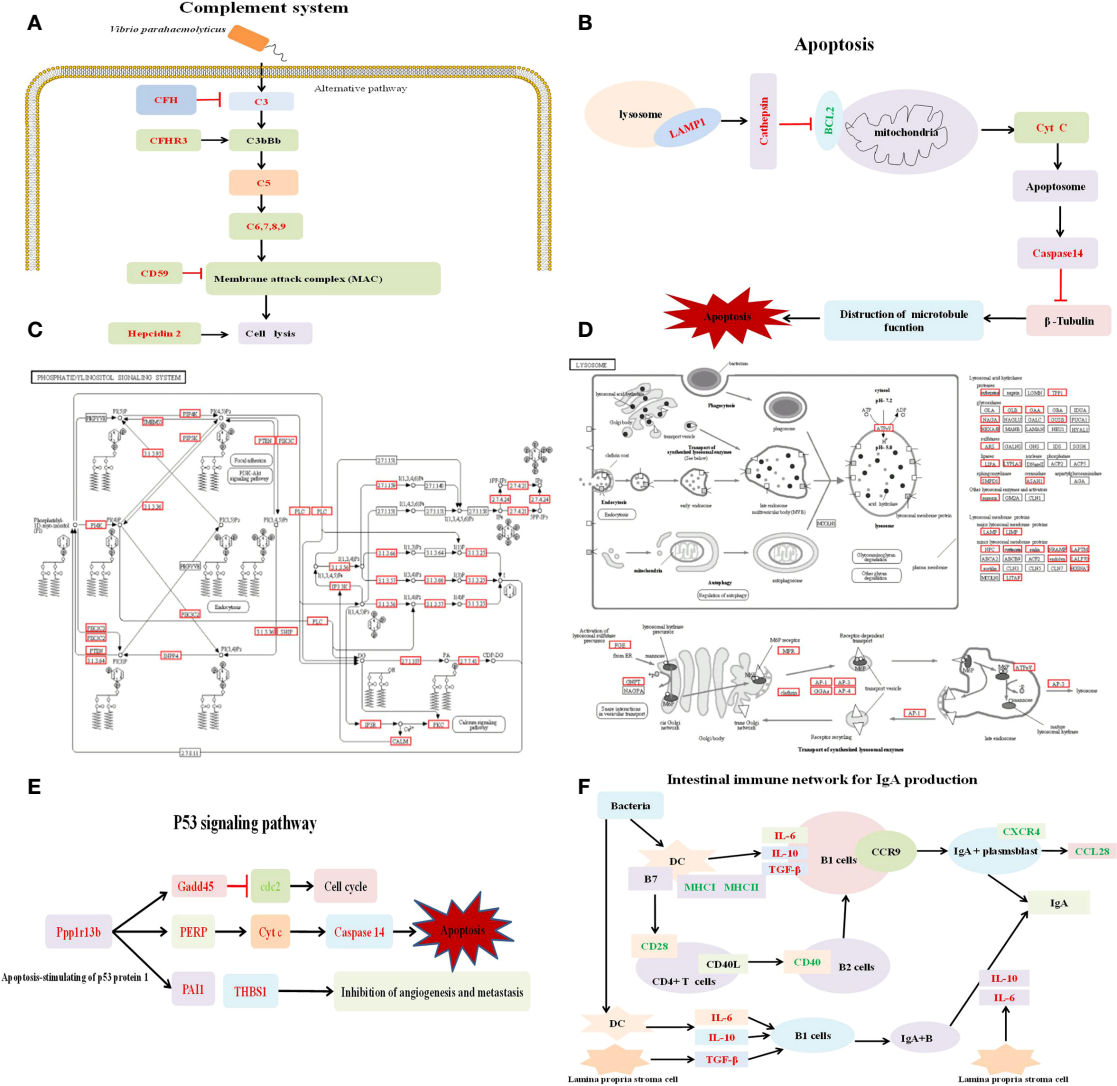
Simplified overview of several pathways. **(A)** Simplified overview of Complement system pathway. **(B)** Simplified overview of Apoptosis pathway. **(C)** Simplified overview of Phosphatidylinositol signaling system pathway. The red box represents significantly enriched target genes. **(D)** Simplified overview of Lysosome pathway. The red box represents significantly enriched target genes. **(E)** Simplified overview of *p53* signaling pathway. **(F)** Simplified overview of Intestinal immune network for IgA production pathway.

The complement system plays an important role in innate immunity, containing three initiation pathways in vertebrates: classical pathway, alternative pathway and lectin pathway. The complement system enhances the ability of phagocytosis of pathogens by promoting inflammation and attacking the plasma membrane of pathogens (47). C1 complex is composed of three subunits: Clq, C1r and Cls3, which can activate the classical pathway (48). Although no change of the gene complement component C1q (*c1q*) was detected in transcriptome sequencing, we found that the expression of complement component C1q receptor (*cd93*) was down-regulated, suggesting that C1q may be involved in the innate immune response of *V. parahaemolyticus* infection. Moreover, the impression of several genes related to the complement system increased, including *c3*, complement component C5 (*c5*), complement component C6 (*c6*), complement component C7 (*c7*), complement component C8 alpha chain (*c8a*), complement component C9 (*c9*), complement factor H related protein 3 (*cfhr3*) and complement factor H (*cfh*). Previous data showed that membrane attack complex (MAC), formed by complement components (C7, C8b, C9), destroyed the cell membrane of the pathogen and eliminated the pathogen by activating the alternative pathway after pathogen infection (49). *C8a*, was cloned from *E. coioides*, and was reported to be effective against *Cryptocaryon irritans* and *Aeromonas hydrophila* (50). As a direct down regulator of the complement classical pathway, CFH is likely to be involved in fine-tuning and balancing the C1q-driven inflammatory processes in autoimmunity and infection (51). Concurrently, the stimulation of LPS (52) and the challenge by *V. alginolyticus* (16) and *P. plecoglossicida* (33, 53) led to activation of the Complement pathway in teleost, which effectively protect the host by promoting the destruction of pathogens. Accordingly, we deduced that after *V. parahaemolyticus* infection, it principally activated the alternative pathway to promote the production of C3, and formed the MAC through the activation of C5, C6, C7, C8 and C9, so as to resist infection (**Figure 7A**).

In order to maintain the normal physiological function of tissue, damaged and dysfunctional cells will be cleared through apoptosis. If the normal cell death process goes wrong, it will bring dramatic effects to the organism. It can be seen that apoptosis is crucial to maintain tissue homeostasis and development. Apoptosis is mainly regulated by external death receptors and internal mitochondria, while the crux to the regulation of mitochondrial intrinsic apoptosis pathway lies in B cell leukemia/lymphoma 2 (BCL-2) family proteins, including pro-apoptotic and anti-apoptotic factors. The down-regulated expression of antiapoptotic protein BCL-2 led to disruption of mitochondrial membrane outer membrane permeability so that proteins normally confined in the intermembrane space spread into the cytosol (54). A pro-apoptotic factor, cytochrome C (Cytc), which binds to apoptotic protease activating factor-1 (APAF1) and triggers the formation of apoptosome. The complex recruits caspase family proteins and induces proteolysis, leading to apoptotic cell death (55, 56). In this study, we found that the expression of *bcl2* was significantly down-regulated, while the expression of *cytc* and apoptosis-related cysteine peptidase 14 (*caspase14*) were significantly up-regulated. These differential genes were mainly enriched in the intrinsic apoptotic pathway, indicating that the intrinsic apoptotic pathway may be efficient to participate in antibacterial immune response (**Figure 7B**).

For miRNA sequencing, a total of 55 significantly different miRNAs were screened, of which 29 up-regulated and 26 down-regulated miRNAs were identified. To further determine the role of miRNAs in the grouper spleen response to *V. parahaemolyticus* infection, the potential target genes of DEMs were predicted and processed for functional annotation. In the KEGG pathway enrichment analysis of target genes of DEMs, the three pathways of “Phosphatidylinositol signaling system”, “Lysosome” and “Focal adhesions” were focused on. Phosphatidylinositol (PI) is widely distributed in eukaryotic cells and modulate many cellular functions, including proliferation, differentiation, apoptosis, metabolism and membrane trafficking. An array of kinases, phosphatases, and lipases acts on PI, resulting in the production of second messengers involved in different cellular processes (57). Many proteins interact with PI to regulate TLR4 signaling pathway (58), which can promote LPS-mediated inflammation. In microglia cell, LPS stimulation can activate PIP5K, which promotes the generation of phosphatidylinositol (PI) 4,5-bisphosphate (PIP2) on the plasma membrane (59). In the present study, 74 target genes of DEMs were significantly enriched in phosphatidylinositol signaling system, such as calmodulin (*calm*), protein kinase C beta type (*prkcb*), inositol monophosphatase 3 (*impa3*), phosphatidylinositol 3-kinase regulatory subunit (*pik3r*), phosphatidylinositol-4-phosphate 5-kinase (*pip5k*), phosphatidylinositol-4-phosphate 3-kinase catalytic subunit (*pik3c*), phosphatidylinositol 4-kinase alpha type (*pi4ka*), myotube related protein 14 (*mtmr14*), etc. (**Figure 7C**). Although toll-like receptor 4 (*tlr4*) has not been identified in grouper until now, it is likely to activate a variety of kinases and proteins to participate in phosphatidylinositol signaling system pathway after recognizing LPS through other receptors, which provides an important reference for grouper against *V. parahaemolyticus* infection.

Lysosomes degrade endocytic extracellular material and intracellular components *via* autophagy (60). Once the lysosomes turn dysfunctional, which will lead to the accumulation of various undigested substances, and eventually bring about serious disease (61). The pathway was believed to be closely related to immune response regulation in *E. coioides* (33). In this study, the Lysosome pathway was also significantly enriched, involving 75 target genes: lysosome-associated membrane protein 1 (*lamp1*), *lamp2*, scavenger receptor class B member 2 (*scarb2)*, cathepsin C/F/H/Z (*ctsc*, *ctsf*, *ctsh*, *ctsz*), lipopolysaccharide induced TNF factor (*litaf*) and so on (**Figure 7D**). This result demonstrates that spleen miRNAs in *E. coioides* may regulate the Lysosome pathway to resist the invasion, and even eliminate the pathogen.

The Focal adhesion pathway also deserves our attention. Focal adhesion (FA) is a junction located below the tight junction of epithelial cells, which connect cells with extracellular matrix by the interaction between integrin and actin. Indeed, pathogenic microbes ensure their uptake, survival and dissemination through the exploitation of FAs (62). A previous study showed that the Focal adhesion pathway was found to be significantly enriched in the omics analysis of infected spleen of *E. coioides* and served as the target of bacterial pathogen (63). *V. parahaemolyticus* was proved to secrete type III effector VopL to disrupt actin homeostasis during infection (7). Meanwhile, the GO term “cell junction” was significantly enriched, indicating that focal adhesion complexes as important cellular structures modified by *V. parahaemolyticus* to help drive infection to the host through the spleen. miRNAs might be of great significance in maintaining the stability of focal adhesions, which serve as momentous signaling hubs within the splenic epithelial cells. In addition, the TCA cycle pathway connected with the Warburg effect was significantly enriched in the KEGG pathway analysis of the target genes of DEMs, which is similar to the KEGG pathway analysis of DEGs. In this case, miRNAs may involve in the immune response against *V. parahaemolyticus* infection by regulating carbohydrate metabolism.

Compared with the analysis of single miRNAs or mRNAs, integrated analysis of mRNA-seq and miRNA-seq contributes to clarify the regulatory role of miRNA-mRNA pairs under the infection. A total of 1427 miRNA-mRNA pairs were screened and then the functional annotation analysis was performed on target DEGs of DEMs. In the KEGG pathways enrichment analysis of miRNA-target pairs, *p53* signaling pathway and Intestinal immune network for IgA production were considered as two crucial immune-related pathways. P53, an activator of apoptosis, can promote the expression of pro-apoptotic genes at the transcriptional level, such as BCL2-associated X (*bax*), p53 upregulated modulator of apoptosis (*puma*), and inhibit the expression of antiapoptotic ones, such as *bcl2* (17). In the enriched *p53* signaling pathway (**Figure 7E**), *miR-144-y*, *miR-378-y*, *novel-m0459-5p* and *novel-m0440-5p* may be the key miRNAs that regulate multiple target genes. The target genes of *miR-144-y* were *cytc* and TP53 apoptosis effector (*perp*), while *miR-378-y* regulated *bcl2* and thrombospondin 1 (*thbs1*), and the target genes of *novel-m0459-5p* were protein phosphatase 1D (*ppm1d*) and *thbs1*. Growth arrest and DNA damage-inducible 45 (*gadd45*) and *caspase14* were targeted by *novel-m0440-5p*. In the existing research, *miRNA-144* regulates *NF-κB* signaling pathway in miiuy croaker *via* targeting *il1b* (64). The overexpression of *miR-378* has been shown to control systemic energy homeostasis and suppress apoptosis initiation (65, 66). These means that *miR-144* and *miR-378* play an important role in innate immune response. For two newly identified miRNAs involved in apoptosis pathway (*novel-m0459-5p* and *novel-m0440-5p*), we can pay more attention to their functions in future research. Notably, SRECI mediated the clearance of apoptotic cells *via* the C1q, and loss of SRECI weakened the uptake of apoptotic cells (67). In current study, the up-regulated expression of scavenger receptor class F member 1 (*srec1*) and *srec2* were found after *V. parahaemolyticus* infection, suggesting that the scavenger receptor F family members play an important role in clearance of apoptotic cells. Herein, we deduced that the miRNAs participate in immune response by regulating the *p53* signaling pathway and cell apoptosis.

Non−inflammatory immunoglobulin A antibodies (IgA), generated in intestinal immunity network, act as the first line to defense the invasion of microorganisms, and promote immune exclusion through microorganisms in mucus (21, 68). The Intestinal immune network for IgA production pathway was identified from the RNA-Seq of infected spleen of *E. coioides* and was one of the major immune response pathways during the defense against bacteria (33). In the Intestinal immune network for IgA production pathway enriched in this study (**Figure 7F**), miRNAs, *miR-378-y*, *miR-7133-y*, and *miR-497-x* regulated *tgfb1*, *mhc1*, *mhc2* and *cxcr4*, respectively. This indicates that *miR-378-y*, *miR-7133-y*, and *miR-497-x* are involved in the Intestinal immune network for IgA production pathway, and the miRNA-mRNA pairs may help *E. coioides* resist *V. parahaemolyticus* infection.

The Cytokine-cytokine receptor interaction, Starch and sucrose metabolism, Glycolysis or gluconeogenesis, ECM-receptor interaction and Regulation of actin cytoskeleton were also significantly enriched, illustrating that differentially expressed miRNA-target pairs regulate these pathways to resist *V. parahaemolyticus* challenge. In short, the results indicate that the interaction between host and *V. parahaemolyticus* is a complex mode and requires in-depth study.

Taken together, the infection of *E. coioides* with *V. parahaemolyticus* led to acute effect. Compared with the control group, the infection could modulate several crucial pathways associating with the Warburg effect and cytoskeleton stabilization, which indicated that the vibrio may exploit the energy of host and disrupt actin homeostasis to promote its proliferation and release of virulence factor. Above all, the infection resulted in innate immune response of host, including activation of a series of immune-related pathways. Understanding the complex interaction pattern will be helpful in resistance of vibrio infection in teleost.

## Conclusion

In conclusion, the study explored the interaction mechanism between *V. parahaemolyticus* and *E. coioides* and indicated that innate immunity plays important roles in response to pathogen infection through mRNA-seq and miRNA-seq analyses as well as the mRNA-miRNA integrated analysis. *Vibrio* may actualize its proliferation and escape immune response by regulating carbohydrate metabolism and actin homeostasis. Moreover, numbers of miRNAs and genes were involved in immune-related pathways including Complement system, Cytokine-cytokine receptor interaction, *p*53 signaling pathway to help host resist infection. This research provides theoretical guidance for *V. parahaemolyticus* disease prevention and control.

## Materials and methods

### Animals and infection of *V. parahaemolyticus*

Healthy *E. coioides* (body length:18.0-22.0 cm, weight: 130 ± 20 g), were purchased from Marine Fishery Development Center of Guangdong Province (Huizhou, China). Then, these fish were acclimatized in a flow-through water system (200 L) for two weeks before the experiment. The seawater was maintained at temperature 28 ± 1 °C and seawater salinity 25–31 during the experiment. The *E. coioides* were fed daily with commercial diet containing 47.0% crude protein (YUQUN OCEAN, China). After anesthesia with MS-222, sixty fish were anesthetized and injected intraperitoneally sub-lethal dose of suspension with 200 μL of 1 × 10^9^ CFU/ml *V. parahaemolyticus* (cumulative survival was counted according to the same steps) while another sixty fish were injected with PBS as a control group. The preparation of *V. parahaemolyticus* was conducted as previously described (12, 69). Briefly, a fish pathogenic strain of *V. parahaemolyticus* previously isolated from a diseased orange-spotted grouper (*E. coioides*) was used, which was confirmed by mass spectrographic analysis and kept in the laboratory. Before challenge experiments, *V. parahaemolyticus* was cultured overnight in marine 2216E broth supplemented with 3.3% NaCl at 28°C with shaking at 200 rpm. After centrifugation, *V. parahaemolyticus* cells were washed and re-suspended three times in phosphate-buffered saline (PBS; pH 7.4) in order to use as an inoculum, and the appropriate bacterial challenge concentration was determined through some pre-experiments (data not shown). Subsequently, samples were taken at different time points of infection (day0, day1, day2, day3, week1, week2), and the spleen collected in each group was used for the preparation of histopathological sections (n=7).

### Preparation of paraffin section

The paraffin sections were prepared according to our previously research (29). Briefly, all spleen samples for examination by light microscopy were dehydrated before paraffin embedding. Five-micrometer slices were cut and then performed with standard hematoxylin-eosin staining. Sealing with neutral balsam (Solarbio, China) and drying overnight at 37 °C, the slices were performed on pathological observation.

### RNA extraction, library preparation and sequencing

Twenty-four hours after *V. parahaemolyticus* challenge, six fish were taken from each group respectively. Then, the spleens of every two fish were mixed into one sample in the infected group and the control. Finally, there were 3 mixed tissue samples in each group. The samples in the infected group were named QS_1, QS_2, QS_3, while the control group was divided into QC_1, QC_2, QC_ 3.

Total RNA was extracted from the samples with a Trizol reagent kit (Invitrogen, USA), after which the integrity was assessed with an Agilent 2100 BioAnalyzer (Agilent Technologies, USA), and the purity and concentration were determined using a Nanodrop 2000C (Thermo Fisher Scientific, USA). The RNA samples with an RNA integrity number (*RIN-value*) ≥ 7 and a 260/280 ratio > 1.8, total concentration > 4 μg were used. After total RNA was extracted, eukaryotic mRNA was enriched by Oligo (dT). Fragmentation was carried out and reversely transcribed into cDNA. Then the purified double-stranded cDNA fragments were end repaired, A base added, after which the fragments were amplified by PCR. The resulting cDNA libraries was sequenced by Gene Denovo Biotechnology Co. (Guangzhou, China) using Illumina HiSeq™ 2500.

MicroRNA-seq libraries were constructed by obtaining small RNA (molecules in a size range of 18–30nt) from total RNA *via* polyacrylamide gel electrophoresis (PAGE). Then the 3’ adapters were added and the 36-44nt RNAs were enriched, after which the 5’ adapters were connected as well. The ligation products were reverse transcribed by PCR amplification and the 140-160bp size PCR products were enriched to generate a cDNA library, which were subjected to Illumina HiSeq™ 2500 by Gene Denovo Biotechnology Co. (Guangzhou, China).

### Total RNA expression analysis

Firstly, the raw sequencing data were filtered by fastp (version 0.18.0) (70) to get high quality clean reads. Then short reads alignment tool Bowtie2 (version 2.2.8) (71) was used for mapping reads to ribosome RNA (rRNA) database and the rRNA mapped reads were removed. The HISAT2. 2.4 (72) was used to align paired-end clean reads to the reference genome of *E. coioides* (the data is not published). For each sample, the mapped reads were assembled by using StringTie v1.3.1 (73, 74) in a reference-based approach. A *FPKM* (fragment per kilobase of transcript per million mapped reads) value was calculated to quantify the expression abundance and variations of each transcription region, using RSEM software (75).

Differential expression analysis was performed by edgeR (76). Basing on the R package, differentially expressed genes (DEGs) were selected as those with false discovery rate (FDR, adjusted *P value*) < 0.05 and absolute fold change ≥ 2. To further determine the biological function of the differentially expressed genes, enrichment analyses were conducted using the Gene Ontology (GO) database (http://www.geneontology.org/) (77) and the Kyoto Encyclopedia of Genes and Genomes (KEGG) database (http://www.genome.ad.jp/kegg/) (78).

### miRNA expression analysis

Raw small RNA sequencing reads were filtered to get clean tags. In order to identify and remove rRNA, snoRNA, snRNA and tRNA, all of the clean tags were aligned with small RNAs in GeneBank database (Release 209.0) and Rfam database (Release 11.0). The clean tags were also mapped to reference genome to remove exon, intron, repeat sequences. Then the remaining sequences were searched against miRBase database to identify known (Species studied) miRNAs. But for unannotated tags, they were aligned with reference genome to identify novel miRNA candidates according to their genome positions and hairpin structures predicting by software MIREAP_v0.2. Ultimately, the novel miRNAs were named miR-x (processed from the 5 ‘-region of pre-miRNA) or miR-y (processed from the 3 ‘-region of pre-miRNA), which distinguished from miR-5p and miR-3p in known miRNAs.

Total miRNA consists of known miRNA and novel miRNA, based on their expression in each sample, a *TPM* (transcripts per million) value was used to calculate and normalize the miRNA expression level. Differential miRNA expression analysis was performed by edgeR software between two different groups or samples. The screening criteria were fold change ≥ 2 and *P* < 0.05. Three sorts of software RNAhybrid (v2.1.2), Miranda (v3.3a) and TargetScan (Version:7.0) were used to predict potential target genes of miRNAs with default parameters, and the intersection of the results were more reliable. To further determine the role of miRNAs in the grouper spleen response to *V. parahaemolyticus* infection, the enrichment analysis of GO terms and KEGG pathways of the predicted target genes were conducted.

### Association analysis and construction of miRNA-mRNA-Pathway network

The integration analysis of total RNA and miRNA was based on the negative correlation between the expression of target gene and specific miRNA, and a software SAS8.1 was used to determine the correlation between miRNA and mRNA expression levels by calculating the Pearson correlation coefficients. The strong correlation was defined with a Pearson’s correlation coefficient > 0.7 and *p* < 0.05. Then the enrichment analysis of GO terms and KEGG pathways were performed on co-expressed negatively miRNA-target pairs (all RNAs were differentially expressed), and a miRNA-mRNA-pathway network was constructed and visualized using Cytoscape software (v3.6.0).

### Validation of the reliability of the sequencing results by qRT-PCR analysis

To validate the reliability of gene expression profiles obtained from RNA-seq results, 25 differentially expressed genes associated with immunity and 13 miRNAs were randomly selected for qRT-PCR verification. The corresponding primer sequences used for validation were listed in **Table 3**. Elongation Factor 1-alpha (*EF-1α*) of *E. coioides* was used as an internal control for the qRT-PCR analysis of DEGs and U6 for DEMs. For DEGs validation, cDNA was synthesized with an appropriate amount of RNA (1 μg) by ReverTra Ace qPCR RT Master with gDNA Remover kit (TOYOBO, Japan), and the qRT-PCR reaction was performed by SYBR^®^ Green Realtime I Master Kit (Roche, Switzerland). According to the manufacturer’s instructions, the reaction mixture was incubated for 10 minutes at 95 °C, followed by 40 amplification cycles of 10 s at 95 °C, 30 s at 60 °C, and 20 s at 72 °C, on a Roche LightCycler 480 Realtime PCR system (Roche, Switzerland). For DEMs validation, cDNA was synthesized by Poly(A) polymerase tailing using a Mir-X**™** miRNA First-Strand Synthesis Kit (Takara, Japan). The preparation of reaction mixture and reaction parameters of further qRT-PCR were as above. All assays were amplified in triplicate wells. The expression of mRNAs and miRNAs was calculated using 2^−ΔΔCt^ method.

**Table 3:** The primer sequences used for qPCR validation.

Primers	Sequences (5’-3’)
**mRNA-seq**	
TLR1-F	CCAGGGTCGCAGAGTCCTATC
TLR1-R	GCCAGCCAAGTTCAGTTTCGT
TLR2-F	AGGGTTCAGAAGGGTTGCTAT
TLR2-R	CAGGAAGGAAGTCCCGTTTGT
TLR3-F	CTGGCTTACTACAACCACCCC
TLR3-R	CAAACTCCCTGCCCTCTTCA
TLR7-F	AAGGTCATAGGATTTGGAGCA
TLR7-R	AGGGAGAAACTGACGGCTTAA
TLR8-F	CGCTTGGACAGTGGGTTTCTT
TLR8-R	GAACTTCGTCCTTCTGGTTCG
IRF3-F	CTGGCTTACTACAACCACCCC
IRF3-R	CAAACTCCCTGCCCTCTTCA
MRC1- F	CAGACGGGAAGACCTGGTTCG
MRC1-R	ATCCAGACTGATTCATAGCGT
LRP1-F	CATTGGCTATTATGGGAGAAA
LRP1-R	TTGTTTCGCAAAATCTTCCAG
LDHA-F	ATCATCCCGAACATCGTCAAG
LDHA-R	GGTGGCGGAAACGGGCAGAGT
JUN-F	CTCTTTTCTGTCGGCTTACGG
JUN-R	CCGCTTGGTACGGGTCTGTCT
FOS-F	CTGACAGCATCAAGTGCCTCC
FOS-R	GCAGAGTTATGAGCCTTGGAT
CCCX1- F	TGGTCCAGCAACCTAACCTTC
CCCX1-R	AGGGGAGGCAGTGGTTGTGAT
CCCX3-F	AAACATCACCGCTCCCATCAT
CCCX3-R	AACACCCAGTCCTGCTTCCAG
C9-F	GTCTTGTCAGGGATCAGTGGG
C9-R	CTGCCTTGCTCATTGCTATCC
TRAF3IP3-F	TTTTCCTTCTTCTTCTCGCTGTG
TRAF3IP3-R	CATGAGATAATCTTCGATACG
MAP3K5-F	CGAGACCAATGAAAATGGCGAC
MAP3K5-R	ACCATCCTGACTGACAGAGCC
C-lectin-F	ATCGCATAACAGAGCCAGAC
C-lectin-R	CAGGGAACATCACTCCAAAC
C7-F	GCTGGAGAAAGGTGAAACGCCGT
C7-R	CCAGTTGTCGTATTTCTCTCCGTT
C3-F	CCTCAACAAGTTTGCTTCC
C3-R	TTATAGTAGCCTGAGTTGATCCGTA
SRECI-F	GCAGGACTTGAACGGCTCG
SRECI-R	CATAATGGCTGTCTTTTGCTGC
SRECII-F	GGGGAGGTCGGCATTTGT
SRECII-R	ATTCACCTCTGGCACGCTCTT
CD163L1-F	GGACAACCGAAAAGTCTAATT
CD163L1-R	CATAGGCTGGGTCATAGTCGG
IL-6-F	CAATCCCAGCACCTTCCAC
IL-6-R	CCTGACAGCCAGACTTCCTCT
PITX3-F	AGGGAAGAACAAGAACAAAACCTG
PITX3-R	TTACATCCCTGGTCGTGCTG
BPI-F	GACACCACATGACAAAGGCAC
BPI-R	ATGTTAAATCCTTGCACCCTCCA
EF-1α-F	GGTCGTCACCTTCGCTCCAT
EF-1α-R	TCCCTTGGGTGGGTCATTCT
**miRNA-seq**	
miR-126-y	TCGTACCGTGAGTAATAATGCA
miR-144-y	GCGGGAGTATAGATGATGTAC
miR-0459-5p	AAGCACCCCTAGTCGTGAGA
miR-0088-3p	CTCAGACTTAGGAAAACTTGC
miR-149-x	GCTCCGTGTCTTCACTCCA
miR-217-x	GGGGTACTGCATCAGGAACT
miR-0053-5p	TTCCGACCATGTTAGCACCA
miR-497-x	GGGGCACTGTGGTTTGTAAA
miR-7133-y	GGGTTTGATACACAGCACAATA
miR-142-x	GGGCATAAAGTAGAAAGCACTA
miR-499-x	GGGTTAAGACTTGTAGTGATG
miR-0440-5p	CAGGTCTCAGGTCTTAGGTC
miR-0468-5p	TGGAAGGCTGAGACACGAC
U6-F	U6 Forward Primer (Takara)
U6-R	U6 Reverse Primer (Takara)
Universal reverse primer	mRQ 3’ Primer (Takara)

### Statistical analysis

The qRT-PCR results were analyzed by SAS8.1 and represented as the mean ± SD. The expression level of mRNA and miRNA were compared between PBS treated group and *V. parahaemolyticus* challenged group. When the *P-value* < 0.05, the groups were considered to be statistically significant.

## Data availability statement

The datasets presented in this study can be found in online repositories. The name of the repository and accession number can be found below: GEO, accession number: GSE207127.

## Ethics statement

The study was reviewed and approved by Animal Care and Use Committee of the School of Life Sciences, Sun Yat-Sen University.

## Author contributions

XQ, conceptualization, methodology, bioinformatic analysis, writing - original draft. YL, data curation, analysis, and writing - original draft. XQ, YL, JX, ND, WL, ZW, YZ, and DL, animal experiment and sample acquisition. HL, YZ, and DL, conceptualization, funding acquisition, resources, supervision, writing – review, and editing. All authors contributed to the article and approved the submitted version.

## Funding

This work was supported by Guangdong Provincial Key R&D Program (2021B0202020002, 2021B0202070002), National Key R&D Program of China (2018YFD0900301), Guangdong Provincial Science and Technology Program (2022A1515010623), the talent team tender grant of Zhanjiang marine equipment and biology (2021E05035), Specific Research Fund of the Innovation Platform for Academicians of Hainan Province (YSPTZX202155) and Innovation Group Project of Southern Marine Science and Engineering Guangdong Laboratory (Zhuhai) (311021006).

## Conflict of interest

The authors declare that the research was conducted in the absence of any commercial or financial relationships that could be construed as a potential conflict of interest.

## Publisher’s note

All claims expressed in this article are solely those of the authors and do not necessarily represent those of their affiliated organizations, or those of the publisher, the editors and the reviewers. Any product that may be evaluated in this article, or claim that may be made by its manufacturer, is not guaranteed or endorsed by the publisher.

## Supplementary material

Supplementary Figure 1 Top 20 GO terms of biological progress category. The abscissa represents the ratio of DEGs to all genes annotated to the given GO term and the vertical coordinate represents the GO terms. The redder bubble indicates more obvious enrichment, with smaller FDR. The larger bubble contains more differentially expressed genes.

Supplementary Figure 2 Length distribution and abundance of small RNAs of the 6 libraries. **(A–F)** Length distribution and abundance of small RNAs from spleen of QC_1, QC_2, QC_3, QS_1, QS_2, QS_3, respectively. The abscissa represents the length of small RNAs, and the vertical coordinate represents the tag counts.
