# Supplementary figures and images for: Integrative analyses of mRNA and microRNA expression profiles reveal the innate immune mechanism for the resistance to Vibrio parahaemolyticus infection in Epinephelus coioides
Source: Front Immunol. 2022 Aug 19;13:982973. doi: 10.3389/fimmu.2022.982973 (PMC9437975; doi:10.3389/fimmu.2022.982973)

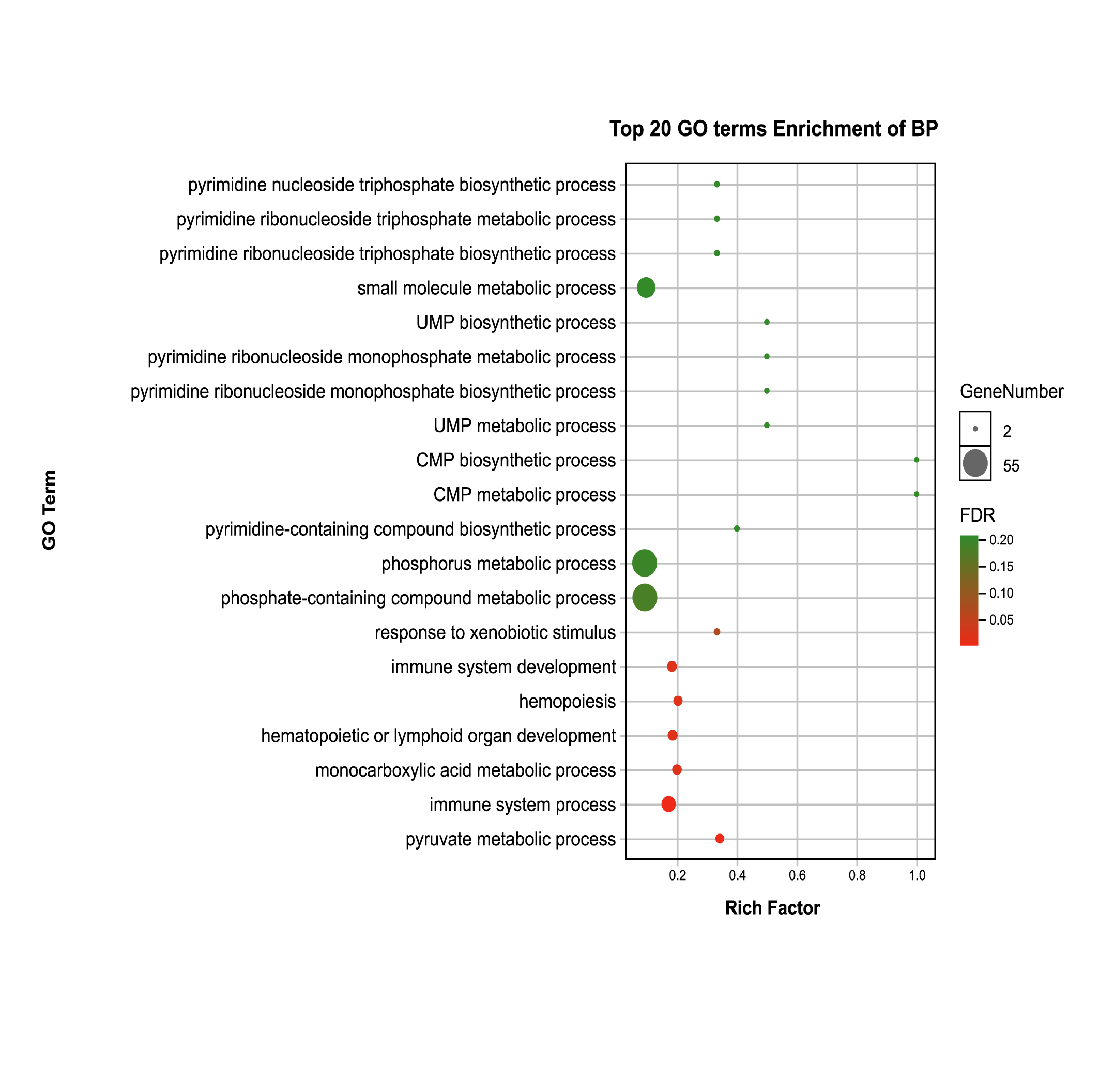

Supplement: Supplementary Figure 1 — Top 20 GO terms of biological progress category. The abscissa represents the ratio of DEGs to all genes annotated to the given GO term and the vertical coordinate represents the GO terms. The redder bubble indicates more obvious enrichment, with smaller FDR. The larger bubble contains more differentially expressed genes. [file Image_1.tif]

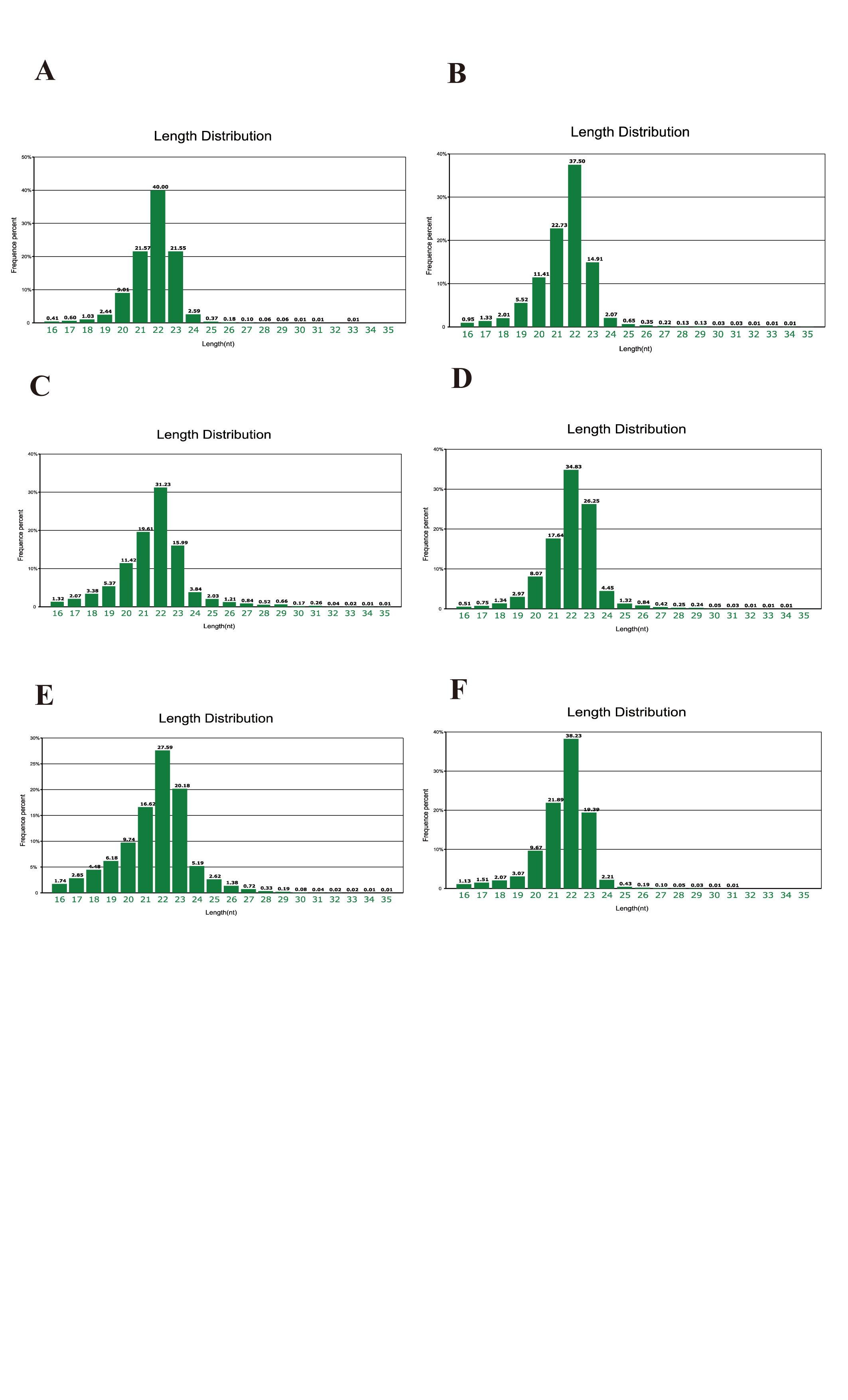

Supplement: Supplementary Figure 2 — Length distribution and abundance of small RNAs of the 6 libraries. (A–F) Length distribution and abundance of small RNAs from spleen of QC_1, QC_2, QC_3, QS_1, QS_2, QS_3, respectively. The abscissa represents the length of small RNAs, and the vertical coordinate represents the tag counts. [file Image_2.tif]
